# Aurora Kinase A expression is associated with lung cancer histological-subtypes and with tumor de-differentiation

**DOI:** 10.1186/1479-5876-9-100

**Published:** 2011-06-30

**Authors:** Marco Lo Iacono, Valentina Monica, Silvia Saviozzi, Paolo Ceppi, Enrico Bracco, Mauro Papotti, Giorgio V Scagliotti

**Affiliations:** 1 Department of Clinical and Biological Sciences, University of Turin, Turin, Italy

## Abstract

**Background:**

Aurora kinase A (*AURKA*) is a member of serine/threonine kinase family. Several kinases belonging to this family are activated in the G2/M phase of the cell cycle being involved in mitotic chromosomal segregation. *AURKA *overexpression is significantly associated with neoplastic transformation in several tumors and deregulated *Aurora Kinases *expression leads to chromosome instability, thus contributing to cancer progression. The purpose of the present study was to investigate the expression of *AURKA *in non small cell lung cancer (NSCLC) specimens and to correlate its mRNA or protein expression with patients' clinico-pathological features.

**Materials and methods:**

Quantitative real-time PCR and immunohistochemistry analysis on matched cancer and corresponding normal tissues from surgically resected non-small cell lung cancers (NSCLC) have been performed aiming to explore the expression levels of *AURKA *gene.

**Results:**

*AURKA *expression was significantly up-modulated in tumor samples compared to matched lung tissue (p < 0.01, mean log2(FC) = 1.5). Moreover, *AURKA *was principally up-modulated in moderately and poorly differentiated lung cancers (p < 0.01), as well as in squamous and adenocarcinomas compared to the non-invasive bronchioloalveolar histotype (p = 0.029). No correlation with survival was observed.

**Conclusion:**

These results indicate that in NSCLC *AURKA *over-expression is restricted to specific subtypes and poorly differentiated tumors.

## Background

*Aurora kinase A *(*AURKA*) is a member of serine/threonine kinase family: homologous to both the *Drosophila aurora *and *Saccharomyces cerevisiae Ipl1 *kinase families. It plays an important role in completing mitotic events such as centrosome separation, bipolar spindle assembly, chromosome segregation and cytokinesis [1]. *Aurora A *expression is cell-cycle regulated. Indeed its mRNA, protein levels and kinase activity are low in the G1/S phase; it accumulates during G2/M and decreases rapidly after mitosis. Aurora A protein is localized in the centrosomes of interphase cells and in the spindle of mitotic cells. Ectopic expression of *Aurora A *leads to an increase in centrosome numbers, causes catastrophic loss or gain of chromosomes, and results in either cell death or survival through malignant transformation [2]. Over-expression of *AURKA *has been detected in many tumor cells and tissues, such as breast, gastric, colorectal, bladder, pancreatic, ovarian, prostate and lung cancers [3-8]. Previous data has pointed out that *AURKA *over-expression is associated with the carcinogenesis and/or drug resistance in many human malignant tumors. Indeed, *AURKA *phosphorylates p53, abrogates both p53 DNA binding and transactivation activities. In such context, *AURKA *overrides the apoptosis and cell cycle arrest induced by cisplatin and γ-irradiation, respectively [9].

*AURKA *over-expression was also correlated with clinical stage and metastasis and its inhibitions to reduce cell invasion in vivo [10,11]. However, *AURKA *expression was involved in the epithelial-mesenchimal transition (EMT) of nasopharyngeal carcinoma. Indeed, the inhibition of *AURKA *suppresses invasion and increases the expression of different epithelial markers [12].

The aim of the present study was to investigate the *AURKA *expression levels in lung tumors and their corresponding morphologically normal lung tissues, obtained from the same resected lobe in patients with early stage NSCLC. Correlation between *AURKA *expression, patients' clinicopathological features and survival was assessed.

## Materials and methods

### Patients and samples

Frozen primary lung tumor and corresponding non-neoplastic lung specimens of 83 consecutive NSCLC patients who underwent radical surgery at the San Luigi Hospital, Division of Thoracic Surgery, between December 2003 and March 2005, were analyzed.

Patients (64 males and 19 females) had a median age of 67 years (range 40 to 82 years) and no patient received either pre-operative or post-operative chemo and/or radio-therapy according to the institutional treatment policy for resectable rescue in those years. Histological examination was performed on formalin-fixed tissues in all cases and tumors were diagnosed and classified according to the WHO classification [13] as follows:

40 adenocarcinomas (ADC); 30 squamous cell carcinomas (SQC); 4 large cell carcinomas (LCC); and 9 bronchiolo-alveolar carcinoma/adenocarcinoma in situ (BAC/AIS). Differentiation grade (grade 1: 16, grade 2: 29, grade 3: 38), pT status (pT1: 7, pT2: 59, pT3: 9, pT4: 8) and pN status (pN0: 52, pN1: 13, pN2: 18) were also recorded. According to the TNM classification for solid tumors [14], 41 cases had a pathological stage I; 15 stage II; 24 stage III; and 3 stage IV. Follow up data was available for all cases. Informed consent was obtained from each patient and the study was approved by the Institutional Review Board of the San Luigi Hospital. All samples were de-identified and cases anonymized by a pathology staff member not involved in the study. Clinical parameters were compared and analyzed through coded data.

### RNA extraction, cDNA synthesis and Qpcr

RNA was extracted from 15-25 mg and 60-80 mg of tumor and normal lung tissue specimens, respectively. Genomic DNA contamination was removed by DNAseI treatment (Promega). TotRNA was then quantified with an Agilent 2100 Bioanalyzer (Agilent Technologies, Palo Alto, CA) and stored at -80°C. Two μg totRNA were retro-transcribed with random hexamer primers and Multiscribe Reverse transcriptase (High Capacity cDNA Archive Kit, Applied Biosystems, Foster City, CA), in accordance with manufacturer's suggestions.

Expression levels of *AURKA *and of reference genes *POLR2B *and *ESD *were evaluated with SYBR technology with optimized PCR conditions and primer concentrations. Primer sequences were as follows: AURKA.FW:GAGATTTTGGGTGGTCAGTAGATG, AURKA.RW:TAGTCCAGCGTGCCACAGAGA, ESD.FW:TGTTGTCATTGCTCCAGATACCA, ESD.RW:CCCAGCTCTCATCTTCACCTTT, POLR2B.FW:CCTGATCATAACCAGTCCCCTAGA,OLR2B.RW:GTAAACTCCCATAGCCTGCTTACC.

Melting curve analysis and efficiency evaluations were performed for all the amplicons. Quantitative PCR (qPCR) was carried-out on an ABI PRISM 7900 HT Sequence Detection System (Applied Biosystems) in 384-well plates assembled by Biorobot 8000 (Qiagen, Germantown, ML). Reactions were performed in a final volume of 20 μl. All qPCR mixtures contained 1 μl of cDNA template, 1Х SYBR Universal PCR Master Mix (2×) (Applied Biosystems). Cycle conditions were as follows: after an initial 2-min hold at 50°C to allow AmpErase-UNG activity, and 10 minutes at 95°C, the samples were cycled 40 times at 95°C for 15 seconds and 60°C for 1 minute. Baseline and threshold for Ct calculation were set-up manually with the ABI Prism SDS 2.1 software.

### Immunohistochemistry

Formalin-fixed paraffin-embedded tissues were cut into 4 μm thick sections and collected onto charged slides for immunohistochemical staining. After de-paraffination and rehydration through graded alcohols and phosphate-buffered saline (pH 7.5), the endogenous peroxidase activity was blocked by incubation with absolute methanol and 0.3% hydrogen peroxide for 15 minutes. Sections were incubated at the optimal conditions with the following primary antibodies:

(1) mouse monoclonal antibody anti-Ki67 (1:300; MIB-1, DakoCytomation, Glostrup, Denmark); (2) Mouse monoclonal AURKA (1:200; H00006790-M01, Abnova, Taipei, Taiwan).

Immunoreaction was revealed by a dextran-chain (biotin-free) detection system (EnVision; DakoCytomation), using 3,3'-diaminobenzidine (DAB; DakoCytomation) as a chromogen. The sections were lightly counterstained with haematoxylin. Negative control reactions were obtained by omitting the primary antibody. Ki67 proliferation index was calculated as the percentage of positive nuclei amongst at least 200 nuclei counted at high magnification in areas of highest labeling.

### Statistical analysis

*AURKA *mRNA Ct values, calculated by Applied Biosystems SDS2.1 software, were normalized by subtraction of the geometric mean obtained between Ct for two internal controls, *POLR2B *and *ESD*, generating ΔCt values. Differential *AURKA *transcript expression between ΔCt values for tumor and corresponding normal tissue samples were evaluated using t-test for paired data and expressed by the formula: ΔΔCt = -(ΔCt cancer - ΔCt normal) corresponding to log_2_[fold change]. Protein and mRNA expression levels have been dichotomized into two groups of "high" and "low" expression using median value as threshold cut-off. For AURKA staining intensity, percentage of cells with nuclear expression and H score (intensity x % cells positive) were evaluated. The association between ΔΔCt and clinico-pathological variables was evaluated using the Kruskal-Wallis test. Overall survival time was calculated from the date of surgery to death or last follow-up date. Cox regression was used in the univariate survival analysis to determine the association of *AURKA *modulation with overall survival. Statistical analysis was performed using R statistical software [15].

## Results

**Figure 1:**
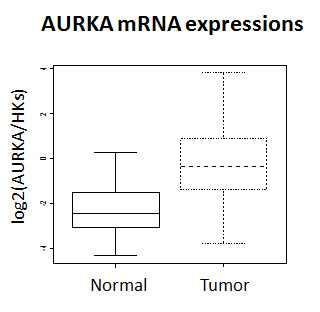
**Increased *AURKA *gene expression in NSCLC patients**. Box plot diagram shows the increased expression level of *AURKA *mRNA in 83 NSCLC respect to the paired non-tumoral tissues.

**Figure 2:**
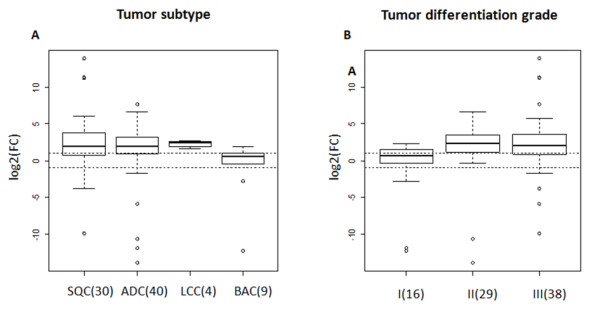
***AURKA *expression patterns in NSCLC correlate with tumor subtype and tumor differentiation grade**. Box plot diagrams showing the modulations of *AURKA *mRNA in 83 NSCLC subtypes specimens. A) *AURKA *was significantly up-modulated in: squamous, adeno and large cells carcinomas (p = 0.029). B) *AURKA *was significantly up-modulated in moderately and poorly differentiated lung cancers (p < 0.01). Dotted lines correspond to a cut-off of ± 2 fold changes (log_2_(FC) ± 1). ADC = Adenocarcinoma, SQC = Squamous cell carcinoma, BAC = Bronchiolo-alveolar carcinoma and LCC = Large cell carcinoma. Values in parentheses indicate the patients' number in each subgroups.

In our NSCLC patients' cohort, we observe a higher *AURKA *transcript level in tumor specimens against the corresponding morphologically normal adjacent lung tissues (Figure.1. p < 0.01, mean log_2_(FC) = 1.5). AURKA mRNA level showed variability according to histological subtypes with the highest expression in squamous cell carcinomas (mean log_2_(FC) = 2.7, p << 0.01) and in large cell carcinomas (mean log_2_(FC) = 2.25, p << 0.01) followed by adenocarcinomas (mean log_2_(FC) = 1.5, p = 0.02) and bronchioloalveolar/in situ carcinomas (mean log_2_(FC) = 0.28, p = 0.4) (Figure.2, panel A). The lowest expression observed in BAC histotypes was significantly different compared to the other tumor subtypes (p = 0.029). Moreover, *AURKA *mRNA was significantly over-expressed in poor (grade III) or moderately differentiated (grade II) lung cancer specimens compared to well-differentiated cases (grade I) (Figure.2, panel B, p < 0.01). No correlation between *AURKA *gene expression and patient's age (p = 0.59), sex (p = 0.12), TNM stage (p = 0.39), or smoking status (p = 0.62) and, with overall survival rates (p = 0.39) was identified.

**Figure 3:**
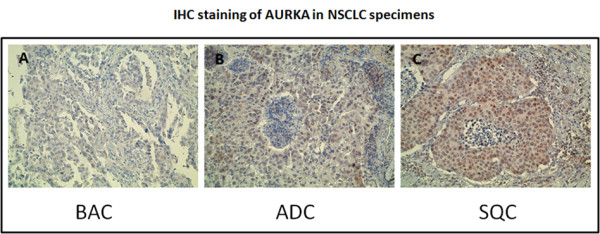
**immunohistochemical detection of AURKA protein in lung cancer**. AURKA was predominantly expresses in nuclear compartment of BAC, ADC and SQC (A, B, C, respectively). Original Magnification 200×. ). ADC = Adenocarcinoma, SQC = Squamous cell carcinoma, BAC = Bronchiolo-alveolar carcinoma.

The AURKA protein expression was investigated by immunohistochemistry (IHC) in 30 NSCLC patients specimens showing nuclear compartment immunoreactivity in 97% samples. Both the associations previously identified between AURKA transcript expression and tumors histological subtypes and differentiation grade were also confirmed at protein level (Table 1 and 2, Figure 3).

**Table 1:** Correlation between AURKA protein expression levels and transcript analysis read-out.

AURKA	% cells with protein expression			H score protein exp		
**mRNA exp tumor**	High	Low		High	Low	
High	10	4	p = 0.03	10	4	p = 0.03
Low	5	11		5	11	

**Table 2:** Correlation between AURKA protein expression levels and transcript analysis stratifying by subtypes and NSCLC differentiation grade.

	mRNA expression		% cells protein exp		H score protein exp	
**Tumor subtype**	High	Low	High	Low	High	Low
ADC	6	6	6	6	6	6
BAC/AIS	1	5	1	5	2	4
SQC	7	5	8	4	7	5
						
**Differentiation grade**	High	Low	High	Low	High	Low
I	2	7	3	6	4	5
II	5	5	3	7	3	7
III	7	4	9	2	8	3

Moreover, since AURKA expression is increased during the G2/M phase cell cycle, we also evaluated the correlation between AURKA expression and proliferation marker ki67. AURKA mRNA expression and ki67 were correlated only in 33% of tumors samples (p = 0.055), and this association was slightly increased for AURKA protein expression (H score: 40%, p = 0.029, % positive cells: 38%, p = 0.04).

*AURKA *expression is involved in the epithelial-mesenchimal transition (EMT) and invasion of nasopharyngeal carcinoma [12]. To test the hypothesis of a similar mechanism in lung cancer we evaluated the effect of AURKA inhibition in NSCLC cell lines (H522, H1299 and Calu1). The FACS analysis reveals that the inhibition of AURKA activity, by the specific inhibitor PHA-739358, slightly increases the expression of E-cadherin (Additional file 1 Figure S1. Panel A), although this effect was transcriptionally independent. Indeed, the *E-Cadherin *gene expression (Additional file 1 Figure S1. Panel B) was unaffected in the H522 cell line by *AURKA *transcript silencing or by AURKA enzymatic inhibition, using the specific inhibitor PHA-739358. Furthermore, the inhibition of AURKA activity does not modify the normal cellular migration of H522 and Calu1 cell lines, while significantly stimulates the motility of high invasive H1299 cell line (*p < 0.05, Additional file 1 Figure S1. Panel C). The experimental procedures utilized in these experiments were illustrate in Additional file 2.

## Discussion

In the present study we evaluated AURKA expression in NSCLC showing that at both transcript and protein levels, the *AURKA *expression was significantly up-modulated in NSCLC tumor samples compared to matched lung normal tissue (p < 0.01, mean log2(FC) = 1.5). The low correlation observed between AURKA expression and ki67 proliferation marker (33-40% with transcript and protein respectively) assert that the *AURKA *up-modulation identified in NSCLC was not only due to a higher proliferation rate but suggests its involvement in cancer pathogenesis. Indeed, we observed a significantly higher *AURKA *transcript expression in poorly and moderate differentiated tumors compared to well differentiated ones (Figure.2, panel B, p < 0.01). Our data is in agreement with a previous report by Xu et al. [8] who identified the AURKA protein over-expression in poorly differentiated lung cancer. Together, this data supports the hypothesis that chromosomal instability associated with progression of lung tumors could be related with *AURKA *deregulation. Xu et al. observed the AURKA protein over-expression in grade III tumors. We also identified the AURKA mRNA over-expression in moderately differentiated tumors. This result may indicate a better sensibility of qPCR analysis respect to IHC for identifying *AURKA *deregulation and the evaluation of AURKA mRNA could be a useful biomarker to identify tumor de-differentiation at early levels.

Our data clearly showed the different histological subtypes of NSCLC exhibited in different levels of *AURKA *modulation ordered from the highest to the lowest as follows: SQC (mean log_2_(FC) = 2.7, p << 0.01), LCC (mean log_2_(FC) = 2.25, p << 0.01), ADC (mean log_2_(FC) = 1.5 p = 0.02) and BAC (mean log_2_(FC) = 0.28, p = 0.4) (Figure.2, panel A). Interestingly, the same histological subtypes ranking was reported also for p53 mutations status [16]. It has been demonstrated that the effect of *Aurora-A *over-expression on tetraploidisation and centrosome amplification depends on the p53 status [17]. Moreover, Tonon et al. suggest a higher grade of genomic instability in SCQ than in ADC [18]. This data, further underlines the tight connection between AURKA over-expression, p53 functions and the genomic instability in NSCLC.

*AURKA *expression is involved in the epithelial-mesenchimal transition (EMT) of nasopharyngeal carcinoma [12] and its inhibition reduces cell invasion in hepatocellular and in head/neck squamous cell carcinoma [10,11]. To test the hypothesis of a similar mechanism in lung cancer we evaluated the effect of AURKA inhibition in non small carcinoma cell lines (H522, H1299 and Calu1).

E-cadherin based junctional complexes keep epithelial cells in a stationary, non-motile state and disruption of this cell-cell adhesion mechanism is a crucial step for tumour invasion. Down-regulation of E-cadherin is one of the main changes occurring in pathological EMTs and causes destabilization of the epithelial architecture [19]. Indeed, E-cadherin acts as a tumour suppressor against invasion and metastasis, and its function is impaired during the malignant progression of most carcinomas including lung cancer [20]. We report that *AURKA *expression/activity in lung cancer cell lines does not regulate the transcriptional level of *E-Cadherin*. This data suggest that *AURKA *expression/activity was not directly involved in lung cancer epithelial-mesenchimal transition. The *E-Cadherin *gene expression (Additional file 1 Figure S1. Panel B) is not increased in the H522 cell line either by *AURKA *transcript silencing, by siRNA technology, or by AURKA enzymatic inhibition, using the specific inhibitor PHA-739358. Moreover, the inhibition of AURKA activity does not modify the cellular migration of H522 and Calu1 NSCLC cell lines, while stimulates the H1299 cell line motility (*p < 0.05, Additional file 1 Figure S1. Panel C). This data suggest that in invasive lung cancer cell lines the cellular motility is not directly dependent on *AURKA *activity and likely its role in invasion could be affected by molecular cancer micro-environment. Further studies are required to investigate if this behavior is shared also by different NSCLC subtypes in vivo and if it may be utilized to select the optimal therapeutic approach of lung cancer subtypes.

## Conclusion

In this study we reported for the first time that NSCLC histological subtypes showed a different degree of AURKA modulation with the highest over-expression observed in SQC and LCC whereas no significant modulation in BAC was reported. We also identified that the AURKA transcript over-expression was significantly associated to tumor de-differentiation, and its activity was not directly associated to either epithelial marker expression or to enhanced cell motility.

Overall, this data supports the emerging network among genomic instability, AURKA over-expression and tumor progression in NSCLC. Further studies are required to elucidate its involvement in chemotherapeutic resistance as its reliability as a putative predictive marker of personalized NSCLC treatments responsiveness.

## Authors' contributions

ML participated in acquiring clinical and laboratory data, data analysis and interpretation, acquiring clinical samples, follow-up clinical information and final writing of the manuscript. VM, SS, PC and EB participated in acquiring clinical and laboratory data, data analysis and data interpretation and drafted the manuscript. MP and GVS participated in study design and coordination, data analysis and interpretation and drafted the manuscript. All authors read and approved the final manuscript.

## Competing interests

The authors declare that they have no competing interests.

## Supplementary Material

Additional file 1 **AURKA expression/activity does not influence migration or Epithelial marker expression in lung cancer cell lines. Figure S1**. *AURKA *expression/activity does not influence migration or Epithelial marker expression in lung cancer cell lines. A) The inhibition of AURKA activity, by the specific inhibitor PHA-739358, weakly increases expression of E-cadherin evaluated by FACS analysis. The highest and the lowest differences between treated and untreated cells were identified in H522 and Calu1, respectively. B) In H522 cell line the inhibition of *AURKA *expression by specific siRNA for different time conditions does not affect the *E-Cadherin *gene regulation (left graph). Moreover, the same results were obtained evaluating *E-Cadherin *transcript expression after treating of the H522 cell line for 24 h with different concentration of Aurora Kinase inhibitor (PHA-739358) (right graph). C) The inhibition of AURKA activity, by the specific inhibitor PHA-739358, does not modify the normal cellular migration of H522 and Calu1, while stimulate significantly the H1299 cell line mobility (*p < 0.05). Click here for file

Additional file 2 **Additional Materials and Methods. Figure S1**. experimental procedure. Click here for file
