# Supplementary figures and images for: Aurora Kinase A expression is associated with lung cancer histological-subtypes and with tumor de-differentiation
Source: J Transl Med. 2011 Jun 30;9:100. doi: 10.1186/1479-5876-9-100 (PMC3148570; doi:10.1186/1479-5876-9-100)

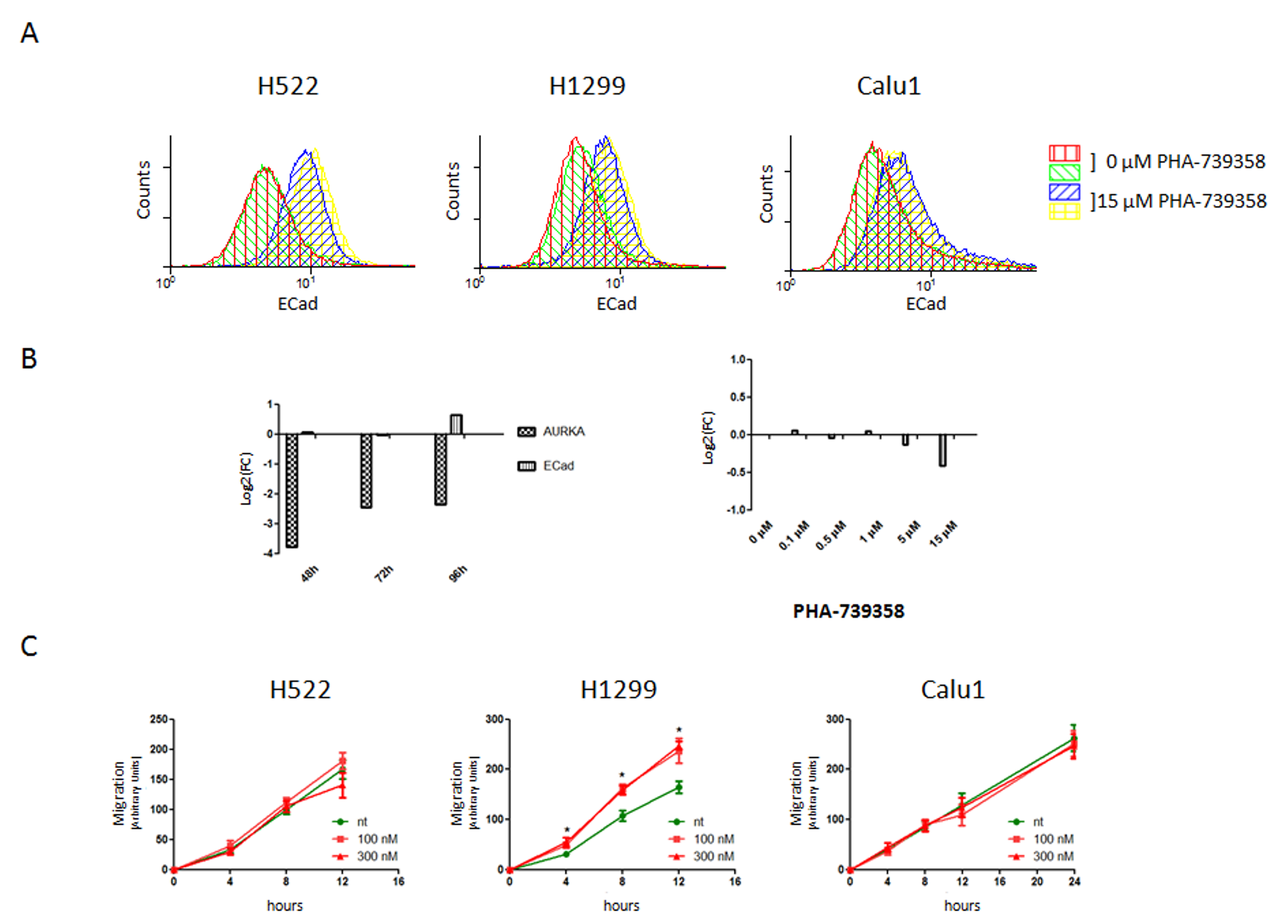

Supplement: Additional file 1 — AURKA expression/activity does not influence migration or Epithelial marker expression in lung cancer cell lines. Figure S1. AURKA expression/activity does not influence migration or Epithelial marker expression in lung cancer cell lines. A) The inhibition of AURKA activity, by the specific inhibitor PHA-739358, weakly increases expression of E-cadherin evaluated by FACS analysis. The highest and the lowest differences between treated and untreated cells were identified in H522 and Calu1, respectively. B) In H522 cell line the inhibition of AURKA expression by specific siRNA for different time conditions does not affect the E-Cadherin gene regulation (left graph). Moreover, the same results were obtained evaluating E-Cadherin transcript expression after treating of the H522 cell line for 24 h with different concentration of Aurora Kinase inhibitor (PHA-739358) (right graph). C) The inhibition of AURKA activity, by the specific inhibitor PHA-739358, does not modify the normal cellular migration of H522 and Calu1, while stimulate significantly the H1299 cell line mobility (*p < 0.05). [file 1479-5876-9-100-S1.TIFF]
