# Supplementary material for: Aurora Kinase A expression is associated with lung cancer histological-subtypes and with tumor de-differentiation
Source: J Transl Med. 2011 Jun 30;9:100. doi: 10.1186/1479-5876-9-100 (PMC3148570; doi:10.1186/1479-5876-9-100)
Supplement: Additional file 2 — Additional Materials and Methods. Figure S1. experimental procedure. [file 1479-5876-9-100-S2.DOC]

**Supporting Supplementary online Material**

**Materials and Methods**

**FACS Analysis.**

ECad staining: 1.5 × 10^4^ of Lung Cancer Cell Lines (H522, H1299, CALU1) treated for 24h with 0 and 15 uM of Aurora Kinase inhibitor (PHA-739358) respectively, were incubated in PBS EDTA 0.1% for 5 min at 37°C. Subsequently detached cells were resuspended in PBS FBS 10% NaN_3_ 1% and stained with primary antibody E-Cadherin antibody [HECD-1] (1:100; ab1416 ABCAM) for 30 min at room temperature. After the cells were stained with secondary antibody Goat anti Mouse-RPE (1:100; Southern BioTech) for 30 min at room temperature, washing twice and analyzed by FACS CyAN adp (Beckman & Coulter) to evaluate E-cadherin expression.

**Cell Treatment, siRNA Transfection and qPCR.**

One day before Treatment, 2 × 10^4^ of Lung Cancer Cell Line H522 for well were seeded in 6-well tissue culture plate in 2 ml fresh complete medium containing 10% FBS. Cells were treated with 0, 0.1, 0.5, 1, 5 and 15 µM of Aurora Kinase inhibitor (PHA-739358) respectively. The plates were further incubated at 37°C for 24 h.

One day before transfection, 2 × 10^4^ of Lung Cancer Cell Line H522 for well were seeded in 6-well tissue culture plate in 2 ml fresh complete medium RPMI-1640 containing 10% FBS. Before transfection, complexes of siRNA/lipofectamine2000 (Invitrogen) reagent were prepared. 40 pMol of siRNA (Scrambled: sc-37007 and ARK-1 siRNA (h): sc-29731; SantaCruz ) and 2 µl of lipofectamine2000 reagent was diluted in 100 µL of OPTIMEM (Invitrogen). The solutions were mixed and left for 20 minutes at room temperature. Before adding the transfection complexes, the complete medium with serum was removed and replaced with 1 ml of serum-free medium RPMI-1640. Then, 100 µL of complexes solution was added per well and the plates were incubated at 37°C. After 6 h of incubation, the transfection medium was integrated with 1 ml of complete medium containing 20% serum and 2x antibiotics. The plates were further incubated at 37°C for 48, 72 or 96 h.

RNA was extracted by TRIZOL (Invitrogen) using standard procedure and analyzed in quantitative PCR. Briefly, expression levels of AURKA and of reference gene POLR2B were evaluated with SYBR technology using optimized condition. Expression levels of *ECad* and of reference gene *POLR2A* were evaluated with Taqman technology using following conditions: All qPCR mixtures contained 1 μl of cDNA template, 1Х TaqMAN Universal PCR Master Mix (2x) (Applied Biosystems) and 1x ECad assay on-demand (CHD1-Hs01023894_m1, Applied Biosystems). Cycle conditions were the same indicated for SYBR green analysis.

**Wound Healing Assay**

After incubation of 16h with RPMI-1640 medium without FBS confluent monolayers of Lung Cancer Cell Lines (H522, H1299, CALU1) were wounded by scratching lines with a yellow pipette tip. The medium was changed with RPMI-1640 10% FBS supplemented with 0, 100 and 300 nM of Aurora Kinase inhibitor (PHA-739358) respectively. The time-dependence of wound closure was compared between treated and untreated cells. Photographs were taken under 50x magnification using phase-contrast microscopy (Axiovert 35; Zeiss ) immediately after wound incision and at later time points.
